# The Impact of Drying and Rehydration on the Structural Properties and Quality Attributes of Pre-Cooked Dried Beans

**DOI:** 10.3390/foods10071665

**Published:** 2021-07-19

**Authors:** Shruti Aravindakshan, Thi Hoai An Nguyen, Clare Kyomugasho, Carolien Buvé, Koen Dewettinck, Ann Van Loey, Marc E. Hendrickx

**Affiliations:** 1 KU Leuven, Laboratory of Food Technology, Department of Microbial and Molecular Systems (M2S), Kasteelpark Arenberg 22, P.O. Box 2457, 3001 Leuven, Belgium; an.nth2412@gmail.com (T.H.A.N.); clare.kyomugasho@kuleuven.be (C.K.); carolien.buve@kuleuven.be (C.B.); ann.vanloey@kuleuven.be (A.V.L.); marceg.hendrickx@kuleuven.be (M.E.H.); 2 Food Structure & Function Research Group, Faculty of Bioscience Engineering, Gent University, 9000 Ghent, Belgium; Koen.Dewettinck@UGent.be

**Keywords:** instant beans, drying, rehydration, volatile analysis, quality

## Abstract

Fresh common beans can be made ‘instant’ to produce fast-cooking beans by first soaking and cooking the beans before drying to create a shelf-stable product that can be rehydrated at the time of use. This study investigated the interplay between the drying process (air, vacuum and freeze drying), the microstructure and functional attributes of rehydrated pre-cooked beans. The microscopic study revealed that the three different drying techniques resulted in distinctly different microstructures, with the freeze drying process resulting in highly porous materials, while the air- and vacuum-dried samples underwent shrinkage. Additionally, the rehydration behavior (modeled using empirical and diffusion models) demonstrates that the high rehydration rate of freeze-dried beans is due to capillarity, while rehydration, in the case of air- and vacuum-dried beans, is primarily diffusion-controlled. Irrespective of the drying technique, the high rehydration capacity supports little to no structural collapse or damage to the cell walls. The color and texture of the rehydrated beans did not differ greatly from those of freshly cooked beans. The total peak area of the volatiles of rehydrated beans was significantly reduced by the drying process, but volatiles characteristic of the cooked bean aroma were retained. This new understanding is beneficial in tailoring the functional properties of pre-cooked dry convenient beans requiring short preparation times.

## 1. Introduction

Despite their nutritional significance, one major aspect limiting the consumer acceptability of beans is their long cooking time and the need for long soaking before cooking [1]. Post-harvest storage of beans under adverse conditions of temperature and relative humidity can lead to (bio)chemical changes resulting in the development of hard-to-cook beans (prolonging their cooking time), or deterioration of their nutritional quality and sensory attributes [2,3]. This creates the need for value-added bean-based convenience products which could be of benefit to consumers and food manufacturers, thereby promoting their consumption [4]. Fresh beans can be made ‘instant’ to produce fast-cooking beans by first soaking and cooking the beans before drying to create a shelf-stable product that can be rehydrated at the time of use. This eliminates the need for soaking at the moment of preparation, with a shorter rehydration time as compared to cooking. Biochemical changes and the associated quality deterioration (i.e., development of hard-to-cook beans) that occur during the storage of fresh beans can be avoided.

Previous research [5,6] in this field primarily focused on reducing drying-induced defects such as splitting and ‘butterflying’ by soaking the beans in a sugar solution, or subjecting them to an alkaline treatment between cooking and drying. However, these processes modify the bean quality characteristics, rendering them unacceptable. A study has also shown that coating with biopolymers before drying considerably reduced butterflying but increased splitting [7]. Attempts [8,9] have also been made to optimize the processing parameters of soaking, cooking and, to a limited extent, drying in producing minimally defective fast-cooking beans. Currently, convective air drying at temperatures < 60 °C is the major drying technique employed, with a mere focus on the rehydration yield. None, however, have explored the impact of different drying processes on the microstructure and its role in influencing the quality attributes of the product obtained (before and after regeneration).

Drying techniques such as freeze and vacuum drying are successfully used in many foods. The influence of these techniques on the structural and quality attributes varies with each having its benefits and limitations. Therefore, this study considered drying techniques such as freeze and vacuum drying, in addition to using the structure–function relation, to unravel the rehydration mechanism and kinetics. 

Rehydration is an important quality indicator as it is a measure of the injury to the product caused by drying. The hysteresis that exists in the product after rehydration is attributed to the product changes that occur during drying [10]. The rehydration rate and extent largely depend on the porosity, structural disruption and surface and capillary structure [11]. In plant matrices, the capacity of cell walls to bind and hold water determines the rehydration and textural characteristic [12]. Beans, unlike fruits and vegetable matrices, possess a larger amount of starch, 25–45% dry matter, which is also major water-imbibing constituent. Therefore, it is hypothesized that in starch-rich matrices, collapse phenomena associated with the gelatinized starch and/or cell wall during drying can impact the rehydration ability and should be taken into account. Other quality aspects of dried beans are reported, but the current work employed targeted approaches, analyzing the color, texture, rehydration and retrogradation of starch, and untargeted volatile headspace fingerprinting approaches to develop a multi-dimensional quality analysis.

The objective of this research is to investigate the interplay between the drying process, bean constituents, microstructure and their relation to the functional attributes. Controlled air, vacuum and freeze drying conditions were used to create pre-cooked dried beans. It is hypothesized that the chosen drying techniques offer significant differences in drying mechanisms and therefore will result in distinctly different process-induced microstructures. This will affect the rehydration kinetics of the materials obtained, which was modeled using empirical models and Fick’s diffusion model to describe rehydration and gain insight into the mechanism. Texture and starch retrogradation are linked to rehydration and the microstructure. To complete the integrated quality evaluation approach, the influence of the drying technique on the color and volatile profile of the rehydrated beans is included.

## 2. Materials and Methods

Fresh red kidney beans were sourced from Belgium. The initial moisture content of the beans was about 13% (method in Section 2.2). A mixture of alkane standards, and 2-heptanon were obtained from Sigma-Aldrich, Belgium. Unless mentioned otherwise, all reagents were of analytical grade. All analyses were performed in at least duplicate.

### 2.1. Preparation of Dried Beans

Red kidney beans were soaked in demineralized water using a ratio of 1:4 (bean/water, *w*/*v*) for 12 h. Soaking water was drained before cooking in demineralized water using a ratio of 1:3 (bean/water, *w*/*v*) at 95 °C for 60 min. Post-cooking, the following drying techniques were employed to create fast-cooking beans:

Freeze drying (FD): The cooked beans were cooled to room temperature and then frozen using liquid nitrogen. Freeze drying (Alpha 2–4 LSC plus, Christ, Germany) was performed under vacuum (condenser temperature of −80 °C, shelf temperature of −20 °C and a chamber pressure of 0.1 mbar) for 12 h.

Convective air drying (AD): The beans were evenly spread on a tray and dried in a cabinet drier at room temperature (25 °C) under an air circulation rate of 130 m^3^/h for 24 h.

Vacuum drying (VD): Drying in a vacuum oven (1445-2, UniEquip GmbH, Planegg, Germany) was performed at a temperature of 50 °C and a vacuum of 0.1–0.2 bar for 16 h.

The drying conditions, including the drying duration for convective and vacuum drying, were chosen based on preliminary experiments which aimed to reduce the defects (splitting and butterflying) generated during pre-cooking and drying. The drying process was deemed complete when the changes in the moisture content were negligible. Lower drying temperatures and long drying processes were most suitable, as also reported by others [4,5]. Given the objectives of this study, namely, the creation of distinctly different microstructures due to differences in drying mechanisms (and afterwards moisture uptake during regeneration), different drying techniques (using conditions with low levels of splitting and butterflying) were used rather than further fine-tuning the processing conditions of a single drying technique. Since the dried products produced by different drying techniques differed in their moisture content, the samples were shortly stored at 100% RH at 4 °C allowing uniform moisture distribution internally [13] until a final moisture content of about 10% (wb) was attained.

### 2.2. Moisture Content

The moisture content was determined gravimetrically by measuring the weight loss of the sample dried overnight in an oven at 103 °C [14].

### 2.3. Rehydration

Rehydration was carried out in distilled water at 70 °C, 80 °C, 90 °C and 100 °C. About 10 g of dried beans was immersed in a water bath with temperature control, and the weight was recorded at regular time intervals until the difference between consecutive weights was insignificant (0.05 ± 0.01 g), representing saturation. No corrections were made to account for the solid loss. The rehydration time corresponds to the time at which the beans attain the saturation weight. The experiment was performed in duplicate. The rehydration potential was defined in terms of yield (*Y*) and relative rehydration moisture content (*RRM*), which were obtained as follows [15]:

(1) $$Y=W_{e}/W_{0},$$

(2) $$RRM=M_{e}/M_{c}$$

The rehydration ratio (*RR*) and moisture ratio (*MR*) are expressed as [16]:

(3) $$RR=\frac{M_{t}-M_{e}}{M_{e}-M_{o}}$$

(4) MR=1−RR

where *M_t_* is the moisture content as a function of time, *t* (min), and *M_e_* and *M_o_* are the equilibrium and initial moisture content of the dried beans, while *M_c_* is the moisture content of cooked fresh beans. *W*_0_ and *W_e_* are the weights (g) originally and at saturation. The moisture contents were expressed in % dry basis, db or g water/g db. Empirical and diffusion models were fit to the rehydration data.

#### 2.3.1. Empirical Models

Empirical models are used because of their suitability and mathematical simplicity. However, they do have little physical meaning and are dependent on the processing conditions [17]. The four most frequently employed models were examined:

Peleg model

(5) $$RR=\frac{1}{M_{e}-M_{o}}\times \frac{t}{K_{1}+K_{2}t}$$

Wbull model

(6) $$RR=1-\exp\left(-{\left(\frac{t}{\alpha }\right)}^{\beta }\right)$$

Exponential model

(7) $$RR=1-\exp\left(-Kt^{n}\right)$$

First-order model

(8) RR=1−exp(−Ht)

The Peleg model is a two-parameter, non-exponential model with *K*_1_ (min % db^−^) being the Peleg rate constant (kinetic parameter related to the reciprocal of the rehydration rate), and *K*_2_ (% db^−^) being the capacity constant. *K*_2_ is related to the saturation moisture content as $M_{e}=M_{0}+1/K_{2}$ [18]. The rest of the models (Equations (6)–(8)) are thin-layer models. The Weibull distribution model is described by two parameters, where *α* is the scale parameter (related to the reciprocal of the rehydration rate), representing the time needed to accomplish 63% of the process, and *β* is the shape factor. In the exponential model, *K* is the rate constant (min^−^), and when *n* = 1, it becomes a first-order model, where the rate constant is represented as *H* (min^−^) [16].

#### 2.3.2. Diffusion Model

Theoretical models employed to describe the water imbibition in foods consider diffusion as the major mechanism. In this context, Fick’s second law can be used to obtain the effective moisture diffusivity which takes into account the internal moisture transfer [19]:

(9) $$\frac{\partial M}{\partial t}=D_{eff}\nabla^{2}M$$

where *M* is the moisture content (db), and *D_eff_* is the effective diffusivity (m^2^/s). If the bean surface is considered as a uniform plate [20] (e.g., when drying a layer of beans), and when the assumptions of negligible shrinkage, negligible external resistance, constant boundary condition, constant diffusivity and temperature are considered, the analytical solution for Fick’s second law of unsteady-state diffusion derived for infinite slabs can be applied [21]:

(10) $$MR=\frac{8}{\pi^{2}}\left[\overset{\infty }{\underset{i=1}{\sum }}\frac{1}{{\left(2i-1\right)}^{2}}\exp(-{\left(2i-1\right)}^{2}\pi^{2}\frac{D_{eff}t}{L^{2}}\right]$$

where *t* is time (s), and *L* is the half-thickness (m).

In using this model to predict the rehydration curves obtained, we estimated both the effective diffusivity (*D_eff_*) and the geometric factor (rather than using 8/π^2^). This factor was estimated as a single bean does not behave as an infinite slab (the bean thickness was used to represent the layer thickness for a layer of single beans).

For the above-mentioned rehydration models, the temperature dependency of the time constants (rate constant or diffusivity) can be represented by the Arrhenius relation (11), with *k_T_* being the rate constant, *k_ref_* being the preexponential factor, *E_a_* being the activation energy (kJ/mol), *R* being the universal gas constant and *T_ref_* being the reference temperature (K). The reference temperature was chosen as 80 °C, roughly the middle of the tested rehydration temperature range.

(11) $$k_{T}=k_{ref}\times \exp\left(\left(\frac{E_{a}}{R}\right)\times \left(\frac{1}{T_{ref}}-\frac{1}{T}\right)\right)$$

The parameters *k_ref_* and *E_a_* were obtained using a single-step approach (substituting Equation (11) into Equations (5)–(8) and (10)) [22]. When applied to the diffusion model, *k_ref_* and *k_T_* correspond to *D_eff_*_0_ and *D_eff_*.

First, for a given drying technique, the rehydration models were applied to the rehydration data to obtain the parameter referring to the time constant. Secondly, the Arrhenius equation (temperature model) was introduced into the drying models, and then, in a single step, we simultaneously estimated the rate constant/diffusivity at the reference temperature (80 °C) and the activation energy.

### 2.4. Microstructure

The microstructure of dried beans and fresh beans was examined using scanning electron microscopy (SEM), Hitachi TM4000 (Hitachi High-Technologies, Tokyo, Japan). A section from the central part of the cotyledon’s cross-sectional axis was viewed at vacuum charged-up reduction (low) at an accelerating voltage of 10 kV at × 200 magnification by backscattered electron (BSE) imaging.

Cryogenic SEM (Cryo-SEM) was employed for the evaluation of cooked and rehydrated beans. A section of the cotyledon was attached to the specimen holder of a CT-1000C cryo transfer system (Oxford Instruments, Oxford, UK) and frozen in slush nitrogen. The sample was sublimed and coated with tungsten before being transferred to the microscopic stage of JSM-7100F SEM (JEOL, Tokyo, Japan) and viewed at an accelerating voltage of 10 kV at × 100 magnification.

### 2.5. Texture

The texture of the rehydrated beans was compared with that of cooked beans. Determination of hardness was by measuring the compression force using a TA-X2i Texture Analyser (Stable Microsystems, Goldaming, UK), as described by Gwala et al. [23]. Half a cotyledon was compressed using a cylindrical flathead aluminum probe to 75% strain at 1 mm/s. The maximum force at compression was defined as the hardness. At least 20 cotyledons per sample were analyzed.

### 2.6. Starch Retrogradation

The degree of starch retrogradation was analyzed using a Q2000 heat flux differential scanning calorimeter with Advanced Tzero™ technology (TA Instruments, New Castle, DE, USA). The instrument was calibrated for temperature and heat of fusion using indium, and an empty pan served as a reference. Samples were weighed into Tzero high-volume aluminum pans with demineralized water to achieve a bean-to-water ratio of 1:3, after which the pans were hermetically sealed. The pans were equilibrated overnight at room temperature before heating from 20 to 100 °C at a rate of 5 °C/min. The temperature of the peak (T_p_, °C), the gelatinization enthalpy (J/g) of fresh beans (ΔH_G_) and the enthalpy of the retrograded starch (ΔH_R_) of the dried beans were determined using the Universal Analysis 2000 software, version 4.5 A (TA instruments). The degree of retrogradation (DR%) was calculated as: $\mathrm{DR}\%={\Delta H}_{R}/{\Delta H}_{G}\times 100$ [24].

### 2.7. Color

The color of dried and rehydrated beans was determined using a Hunterlab ColorQuest colorimeter (Hunterlab, Reston, VA, USA). Calibration was performed using a standard white tile, and the color values (represented in the CIELAB color space) were measured as L* (lightness/darkness), a* (redness/greenness) and b* (yellowness/blueness). The total color change (ΔE*) was then determined from the following equation:

(12) $$\Delta E^{*}=\sqrt{{\left(L^{*}-L_{0}^{*}\right)}^{2}+{\left(a^{*}-a_{0}^{*}\right)}^{2}+{\left(b^{*}-b_{0}^{*}\right)}^{2}}$$

where subscript ‘0’ refers to the color of the control sample (fresh beans in the case of dried beans, and cooked beans for rehydrated beans), and ΔE* and ΔE_r_* are the color change of dried and rehydrated beans, respectively [25].

### 2.8. Analysis of Volatile Compounds

#### 2.8.1. Headspace-Solid-Phase Micro-Extraction-Gas Chromatography-Mass Spectrometry (HS-SPME-GC-MS) Analysis

The procedure described by Chigwedere et al. [26] was followed. Headspace analysis was performed using a GC system (7890 N, Agilent Technologies, Diegem, Belgium) coupled to a mass selective detector (MSD) (5977 N, Agilent Technologies, Diegem, Belgium) and equipped with a CombiPAL autosampler (CTC Analytics, Zwingen, Switzerland). The dried beans were rehydrated with water (1:3 ratio, *w*/*v*) in Duran bottles at 100 °C for their respective rehydration times. Immediately thereafter, the bottles were immersed in an ice bath to halt cooking and condense the volatiles. The contents were then homogenized using an IKA T25 digital ultra-turrax (Janke and Kunkel, Staufen, Germany) at 8000 rpm on an ice bath. Six repetitions were created for each sample. Amounts of 4 g of the sample and 4 mL of saturated sodium chloride solution were added to 20 mL amber-color vials (VWR International, Radnor, PA, USA) with 2-heptanon as an internal standard.

The samples were incubated at 40 °C for 30 min with agitation, after which the volatiles were extracted using an HS-SPME fiber coated with 30/50 μm divinylbenzene/carboxen/polydimethylsiloxane (DVB/CAR/PDMS) (StableFlex, Supelco, Bellefonte, PA, USA) at 40 °C for 10 min. The volatile sample was injected using a splitless mode and separated on a polar HP INNOWax capillary column (60 m × 0.25 mm × 0.25 μm, Agilent Technologies, Santa Clara, CA, USA) with helium as carrier gas at 1.5 mL/min. The oven was programmed at 40 °C for 2 min, after which the temperature was elevated to 80 °C at 3 °C/min and maintained for 1 min, where, thereafter, it was ramped again to 220 °C at 6 °C/min, with a holding time of 2 min. A final ramp at 50 °C/min to 250 °C was performed, and, immediately after, the oven was cooled to its initial temperature. The mass spectra were obtained at 70 eV, with the ion source and quadrupole at 150 °C and 230 °C, respectively, while a mass-to-charge ratio scanning range of 35–400 at 3.8 scans/s was employed. A mixture of n-alkanes (C_8_–C_22_) was used as standards to obtain a retention index standard (calibration data).

#### 2.8.2. Data Processing and Multivariate Analysis

The chromatograms were analyzed using automated a mass spectral deconvolution and identification system (AMDIS) (Version 2.72, 2014, National Institute of Standards and Technology, Gaithersburg, MD, USA) to obtain pure component spectra. Moreover, peak filtering and alignment were performed using Mass Profiler Professional (MPP) (Version 12.0, 2012, Agilent Technologies, Diegem, Belgium) [26]. The total peak area of the volatiles of each of the rehydrated samples was obtained as the average of the repetitions and was compared among the different samples.

Multivariate data analysis was performed using SOLO (Version 8.7.1, 2020 Eigenvector Research, Wenatchee, WA, USA). To compare the effect of different drying techniques on the rehydrated samples, partial least squares discriminant analysis (PLS-DA) was implemented with volatile compounds as *X* variables, and rehydrated air-dried, freeze-dried, vacuum-dried and cooked (control) samples as categorical *Y* variables. The model complexity was based on the lowest number of latent variables (LVs), resulting in class separation. The grouping and/or separation of the classes was depicted using a bi-plot, constructed using OriginPro 2020 (Origin Lab Corporation, Northampton, MA, USA). The volatiles important for class separation, i.e., discriminant compounds, were identified using variable identification (VID) coefficients [27]. Variables with a VID of 0.8 and above were considered. A positive VID corresponds to a higher concentration of the discriminant compound in the certain treatment class compared to the others and vice versa. The identification of the discriminant compounds was by comparing the deconvoluted mass spectrum with the reference mass spectra from the NIST spectral library (NIST14, version 2.2, National Institute of Standards and Technology, Gaithersburg, MD, USA). Confirmation was made by comparing the retention index (RI) with available literature data [28].

### 2.9. Statistical Analysis

The parameters of the rehydration models were estimated by non-linear regression using SAS version 9.3 (SAS Institute Inc., Cary, NC, USA), where the details can be found in Section 2.3. The concordance between the experimental and calculated data was evaluated using the coefficient of determination (R^2^), chi-square (χ^2^) and root mean square error (RMSE). Additionally, the parity and residual plots were also visually inspected (data are shown in Supplementary Materials). Statistical significance between treatments was analyzed using one-way ANOVA, and Student’s *t*-test was used for the comparison of means at the 95% significance level (*p* < 0.05).

## 3. Results

### 3.1. Microstructure of Dried Beans

**Figure 1:**
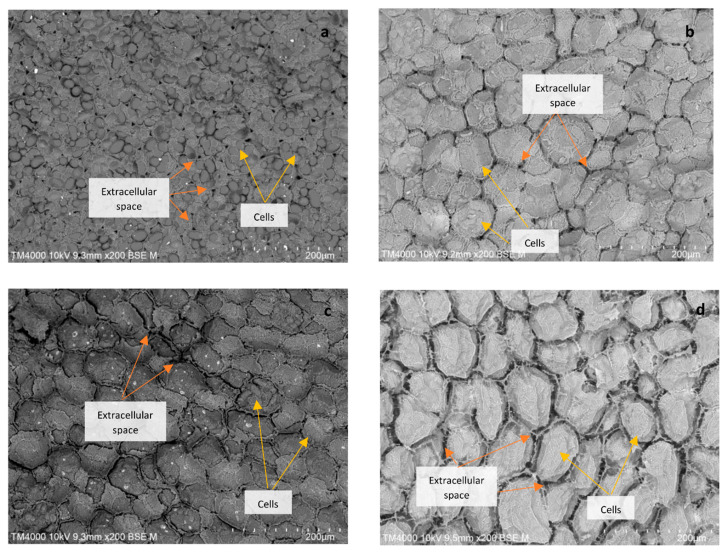
SEM micrographs of fresh (**a**), air-dried (**b**), vacuum-dried (**c**) and freeze-dried (**d**) beans. Scale bar: 200 µm.

The microstructural evaluation (Figure 1) depicted differences between the cells of fresh beans and dried beans. Fresh beans displayed well-organized cellular structures (including starch granules) with distributed small intercellular spaces. The individual starch granules were no longer visible in the cooked dry beans. Among the AD and VD beans, there were no pronounced differences between the cell sizes and spacings, although they were larger than for fresh beans due to cooking phenomena (i.e., starch gelatinization). At a macro- and microscopic scale, AD and VD beans showed considerable shrinkage (compared to cooked beans) upon drying; however, the intercellular spaces were still maintained. The spaces were also smaller, and the cells were closely packed, as compared to FD bean cells. The larger size of FD cells is partly because the water is replaced by ice in the matrix during freezing, leading to an increase in the volume due to the lower density of ice compared to water [29], and mainly due to the absence of shrinkage (collapse) during the freeze drying process. The ice is sublimated and replaced by air, leading to a texturally ‘fragile’ product.

### 3.2. Rehydration

#### 3.2.1. Effect of Different Drying Processes on Rehydration

**Figure 2:**
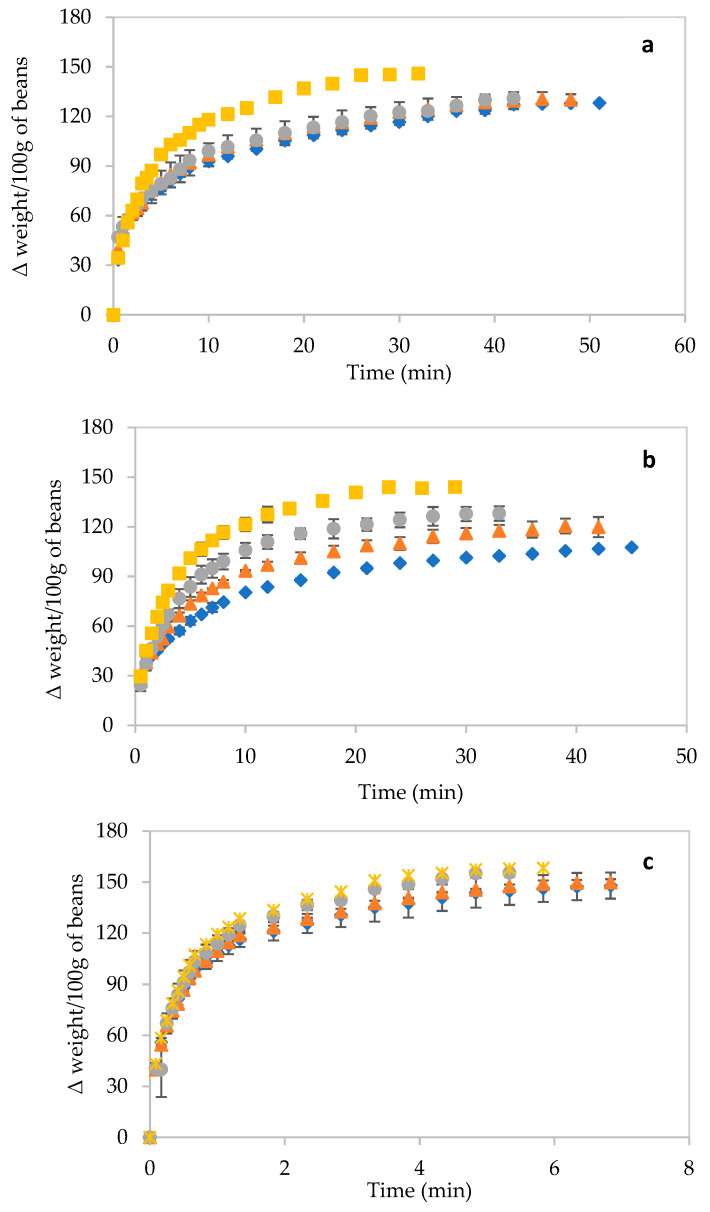
Rehydration profiles of air-dried (**a**), vacuum-dried (**b**) and freeze-dried (**c**) beans at 70 °C (◆), 80 °C (▲), 90 °C (●) and 100 °C (■).

In this study, hot air drying was not considered as research has demonstrated that drying at high temperatures can lead to collapsed structures, with the products possessing a poor rehydration ability [11]. Therefore, AD at ambient temperature was chosen instead. VD can overcome the limitation of air drying as the vacuum enables the use of a lower drying temperature, rendering it suitable for heat-sensitive materials and requiring a lower drying time than air drying. FD is considered a superior technique as the products have a good rehydration ability and sensory qualities owing to the low drying temperature and high porosity of the dried products [30]. The rehydration profile of dried beans at different temperatures of rehydration (70 °C, 80 °C, 90 °C and 100 °C) is depicted in Figure 2.

Irrespective of the temperature of the rehydration medium, rehydration was faster in the initial phase of the process and then slowed down, finally reaching an equilibrium. Some leaching of the solutes might also occur simultaneously. The rehydration of FD beans was 9–10 times faster than that of AD and VD beans, which exhibited almost similar rehydration times. This difference in the rehydration rate is attributed to differences in the porosity of the structures obtained after drying. Freeze drying leads to the creation of multiple interconnected open pores, while in contrast, the lower rehydration rate in AD and VD samples manifests in closed or blind pores. Together with their dense and shrunken structure, this results in lower rehydration rates. Increasing the rehydration temperature resulted in a higher rehydration ability (related to *Y* and RRM), with a decreased rehydration time. This effect was prominent in vacuum-dried beans, but also, to a lesser extent, in AD and, in particular, in FD beans. The rehydration ability depends on the internal structure as well as composition, with starch and cell wall components being the major water-retaining components. The drying technique did not affect the rehydration ability because the cellular structure of the dried beans was not destroyed. The cell configuration would most likely possess intact cell walls with sustained intercellular spaces. This also strongly suggests that cell wall reconstitution was most likely not very much hindered.

#### 3.2.2. Modeling of Rehydration

##### Empirical Models

The parameter estimates for the four empirical rehydration models (applied to each rehydration curve) are presented in the Supplementary Materials (Table S1). The Peleg model has been frequently used to investigate the water uptake by legumes during soaking [31,32,33]. Peleg’s *K*_1_ decreased with the increase in the rehydration temperature for VD and AD beans, indicating a higher rate of rehydration with temperature, but it was nearly constant for FD beans. Irrespective of the rehydration temperature, *K*_1_ was also the lowest for freeze drying, as evident by its highest water absorption rate. Peleg’s *K*_2_, associated with the water absorption capacity, only decreased slightly with the temperature. On the other hand, among the drying techniques, Peleg’s *K*_2_ was somewhat lower for FD beans. This difference is due to the effect of drying on the material properties and thereby the rehydration ability.

The Weibull parameter *α* was also related to the water absorption rate and displayed similar trends to Peleg’s *K*_1_. The smallest increase in the temperature was observed for FD beans. The slight increase in *α* with the temperature of FD beans might indicate collapse during rehydration. Over the different drying techniques and drying temperatures, *β* values ranged from 0.487 to 0.724, and attempts have been made in the literature to relate *β* to different mechanisms of moisture uptake. The values obtained in this study do not correspond to those of pure Fickian diffusion (*β*~0.8) [34]. Besides that, the exponential and first-order models were also investigated. The exponential model is mathematically identical to the Weibull model, with *n* = *β*, and *k* = (1/*α*)^β^. In both cases, the kinetic parameters increased with increasing temperature, with the first-order model performing rather badly (lower R^2^ and non-random error distribution).

The overall influence of the temperature was estimated by calculating the activation energy of the kinetic parameters. The *E_a_* based on the kinetic parameters of the best fitting models (Table 1) was the highest for VD beans, followed by AD and FD beans. The results obtained in this study are lower than the reported data [35] for AD common beans.

Each of the models can fit the rehydration well, but the Weibull model (and the exponential model, being mathematically identical), followed by the Peleg model, displayed the best fit in terms of the random distribution of errors (Figure S1a), the highest R^2^ and the lowest RMSE and χ^2^ value (Table S1). Despite describing the rehydration process appropriately, the parameters of the empirical model provide little insight into the complex mechanism of rehydration.

##### Diffusion Model

In this study, the diffusivity was obtained from the overall experimental curve, and describing separate processes (i.e., the initial and final stages of rehydration) was deemed unnecessary. The parameter estimates (the effective diffusivity and geometric factor) at different rehydration temperatures for the different drying methods are shown in Table S2. The estimated geometric factor at all rehydration conditions varied from 5.531 to 6.534. For an infinite slab and sphere, the theoretical geometric constant equals 8 and 6, respectively [20]. As a result, the instant bean should be considered a spherical object rather than an infinite slab. The diffusivity value of beans dried by freeze drying was 10 times higher than that of AD and VD beans. This relates strongly to the rehydration time which is of the same order for AD and VD, but ten times higher for FD. The high apparent diffusivity of FD beans could be attributed to their highly interconnected porous structure. For each drying process, diffusivity also increased as a function of the temperature due to the increased inner migration of water. The temperature dependence of *D_eff_*, described by the Arrhenius equation was used to calculate the activation energy. The results (geometric factor, *D_eff_*_0_ and *E_a_*) are reported in Table 1, together with the values obtained for the empirical models. A higher *D_eff_* and lower *E_a_* were obtained in this study compared to those of other studies [36] for the rehydration of beans dried by hot air drying. The model performance (parity and residual plot in Figure S1b) reflects the adequacy of the diffusion model in explaining the rehydration characteristics of the dried beans, especially in the case of air- and vacuum-dried beans.

Based on the apparent diffusion coefficients and activation energies obtained, we can hypothesize on the moisture transfer mechanisms involved in rehydrating beans dried by the different techniques. The high rapid moisture uptake of water by FD beans suggests that water is not just adsorbed quickly but is also available to be transported through the intercellular spaces by capillary transport and can rapidly diffuse across the cell wall and membrane into the cell. The uniform honeycomb porous structure of FD beans provides strong evidence that capillarity is the major mechanism. On the other hand, the rehydration rate of the AD and VD beans was much slower (lower apparent diffusion coefficients), and their temperature dependence was more pronounced (particularly for VD beans), suggesting that diffusion (rather than capillary transport) plays a major role. The rehydration times are only slightly lower than the cooking times of fresh beans. These drying processes lead to a shrunken structure with conserved intercellular spaces, but not open pores, with moisture transport thus being governed by diffusion. Our conclusions are in line with previous studies suggesting that the rate-limiting step for rehydration is adsorption and the subsequent internal transfer of moisture [37]. Capillarity is the major model of water transport in porous foods, with porosity determining the rehydration potential, while diffusion is the major model of water transport in the case of solid low-porosity foods [38].

### 3.3. Microstructure of Rehydrated Beans

**Figure 3:**
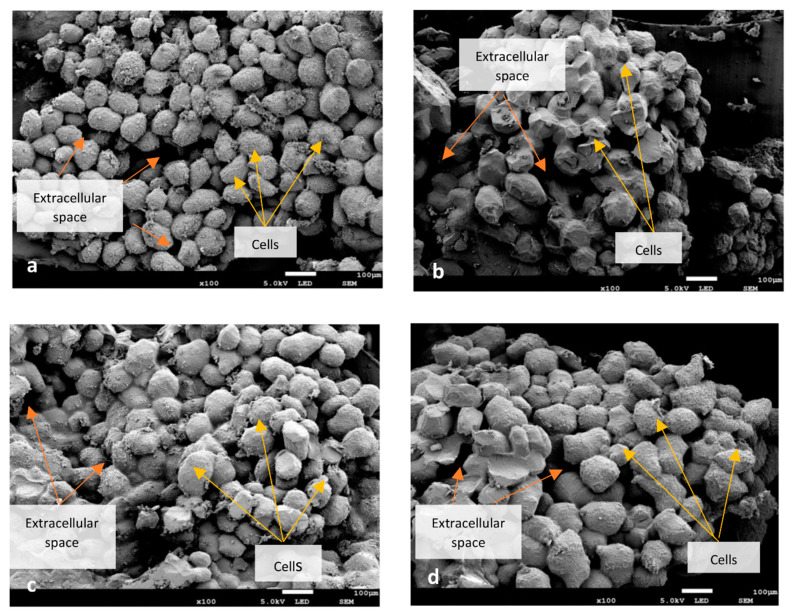
Cryo-SEM micrographs of cooked beans (**a**) and rehydrated air-dried (**b**), vacuum-dried (**c**) and freeze-dried (**d**) beans. Scale bar: 100 µm.

The microstructural similarity between rehydrated dried beans and freshly cooked beans is apparent in Figure 3. Swollen cells with intercellular spaces filled with leached solutes (although not to a great extent) are observed in all rehydrated dried beans. Damage to the cellular structure caused by the pressure of vaporized water within the cell during drying or by the ice crystals formed during freezing led to the presence of leached solids in extracellular spaces. In a similar study on chickpeas [15], rehydrated chickpeas, dehydrated by a higher level of microwave power, displayed similar results, suggesting that a higher intensity of processing can damage the cell wall. On the other hand, cooked beans possessed lower leached solids, as highlighted by the clear extracellular spaces and less dense structure when compared with rehydrated beans.

### 3.4. Starch Retrogradation

Gelatinization and retrogradation are primary processes that shape the characteristic properties of starch, which consequently exert a great impact on the functional properties of starch-based products. These behaviors decide the quality attributes such as texture, consistency, mouthfeel, consumer acceptability, nutritional value and storage stability of the final products [24]. The Y values (Table 2) of dried beans indicate that they are capable of imbibing about 2–2.5 times the amount of water, and therefore the solid-to-water ratio used in this study was considered sufficient for the complete gelatinization of starch. The thermal analysis of the dried samples after rehydration revealed that the starch in dried samples was retrograded. The retrogradation of starch took place during drying and might have continued during moisture equilibration at 4 °C. The results (Table 2) indicate that the gelatinization temperature and enthalpy of retrograded starch are lower as compared to the native starch in the beans. Among the dried beans, the VD beans had a significantly (*p* < 0.05) higher melting temperature than AD and FD beans, which were almost similar, related to the higher drying temperature employed during vacuum drying. On the other hand, the degree of retrogradation was significantly (*p* < 0.05) higher in AD. The drying conditions and/or the longer equilibration time required for the AD beans lead to higher amounts of retrograded starch. Although differences in retrogradation are found in the different drying techniques, the starch will show complete gelatinization after regeneration of the beans at temperatures above the melting temperature (regeneration temperatures of 70 °C and higher were used).

### 3.5. Color

**Figure 4:**
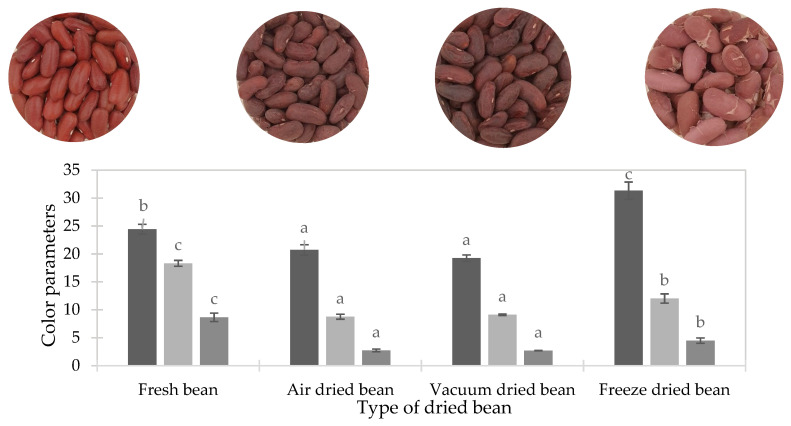
Color parameters L* (■) a* (■) and b* (■) and visual appearance of dried beans and fresh beans. Parameters not sharing the same letters are significantly different (*p* < 0.05).

Color is an important sensory attribute, and a lower value of L* and an average total color change are targeted as good quality indicators during the drying process. Irrespective of the drying technique, the dried beans exhibited color changes (lower L*, a* and b*) as compared to the fresh beans (Figure 4). AD and VD beans were darker (lower L*), with no significant differences between them, while the ones obtained by freeze drying were significantly (*p* < 0.05) lighter. However, ΔE* did not differ among the dried beans (Table 2). This suggests that cooking followed by drying merely led to a concentration of pigments in the dried beans, and the change in brightness was associated with the removal of water. In the case of rehydrated samples, lightness was comparable between rehydrated AD and FD beans and the freshly cooked beans, while it is marginally lower in rehydrated VD beans. b* was significantly lower in rehydrated VD beans, suggesting the loss of color pigments during drying, but it was near minimal. ΔE* was lower for rehydrated samples compared to dried samples, with ΔE* being the lowest for rehydrated air-dried beans, which is a result of the mild processing intensity. Since the extent of ΔE* upon functionalization of the beans did not manifest at the same level upon rehydration, it marks the reversibility of the color change, with rehydrated beans being very close to their freshly cooked counterparts.

### 3.6. Texture

Food texture and structure are inextricably linked and can depend on the plasticizing effect of water and the damage caused by processing. The average hardness (Table 2) of rehydrated AD and VD beans did not differ significantly from cooked fresh beans, while that of rehydrated FD beans was significantly (*p* < 0.05) lower but was in the palatable range. Cryo-SEM micrographs of rehydrated FD beans confirm this textural observation, depicting higher cellular damage owing to the formation of ice crystals during freezing, leading to higher extracellular leached solids. Once the freeze-dried material is rehydrated, it quickly loses its elasticity of cellular texture and becomes more viscous. In contrast, AD and VD beans still retain their viscoelastic behavior after rehydration due to their strong, dense structure as a result of the considerable shrinkage [12]. This is also related to RRM (Table 2), which was the highest for rehydrated FD beans, implying its ability to absorb more water which, in turn, leads to a lower hardness.

### 3.7. Untargeted Volatile Fingerprinting

The flavor profile of rehydrated dried beans was studied at their corresponding rehydration times and compared to that of cooked fresh beans. In this study, the volatile profile of non-cooked beans was not considered as it is the cooked/rehydrated beans that are of critical importance from a sensorial point of view. A significant reduction (*p* < 0.05) in the total peak area (results not shown) was observed between the rehydrated dried beans and the cooked beans, with AD being significantly higher than FD and VD (not different). The loss of volatiles occurs during cooking and drying, both being key steps in the process.

**Figure 5:**
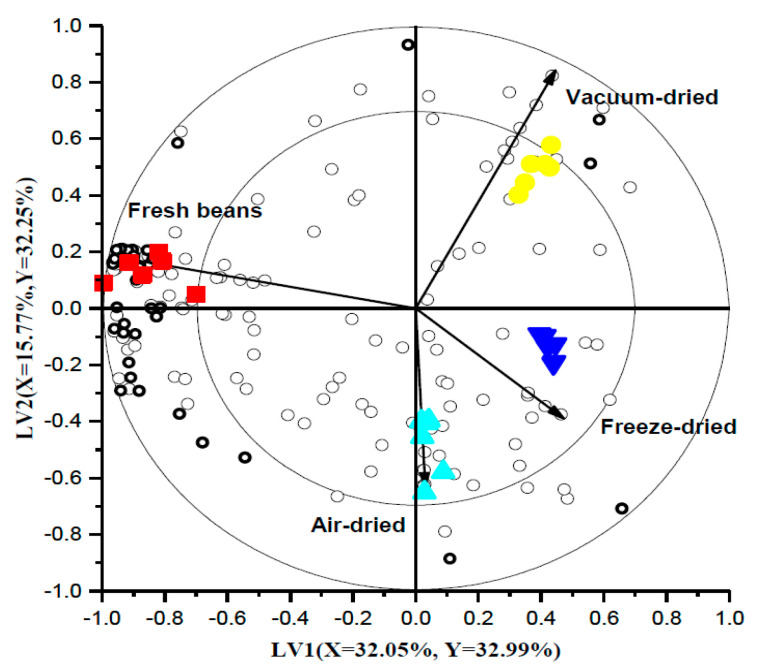
PLS-DA bi-plot of the headspace fraction of cooked (■) and rehydrated air-dried (▲), vacuum-dried (●) and freeze-dried (▼) beans. The open circles are the volatile compounds among which the discriminant compounds are marked in bold. The correlation loading for the *Y* variable is represented by the vector. The percentages of *X* and *Y* variance explained by each latent variable are indicated on the respective axes.

A PLS-DA model with three LVs was applied to understand and compare the volatile fractions between the cooked and rehydrated dried beans, in which LV1 and LV2 were chosen to build a bi-plot (Figure 5). The grouping and/or separation between the treatments was visualized with closer classes, being considered similar and vice versa. In this regard, rehydrated AD and FD beans are close, meaning they have similar volatile characteristics and are separated from cooked and VD beans. This is because AD and FD beans were not exposed to high temperatures, as happened with VD beans. The inner and outer ellipses in the bi-plot represent a correlation coefficient of 70% and 100%, respectively. Moreover, the compounds located on the vectors (correlation loading for *Y* variables) pointing towards each class are positively correlated with that class, with the vector length and position indicating the performance of the model.

For quantitative comparison of the discriminant power of each volatile compound for a corresponding sample, VID coefficients were first calculated, and only the volatiles with an absolute value of more than 0.8 (ǀVIDǀ > 0.8) were chosen as discriminant compounds. Table 3 presents all volatiles that met the requirements in decreasing order of VID. Most discriminant volatiles in dried beans were associated with a positive VID, indicating their formation or presence at significantly increased amounts post-drying. Drying resulted in a significant decrease in alcohols and ketones. Moreover, furan derivatives and sulfur-containing compounds did not present as major discriminant compounds for the dried beans even though they are associated with high-temperature processing [39]. However, the formation or increase in the content of other aldehydes, alcohols, alkanes, and organic acids in the dried beans can also be seen.

Alcohols such as 2-methylpropanol, ethanol and 2-hexanol were detected in abundant amounts in freshly cooked beans. While two alcohols, 2-phenyl-ethanol and 3-methoxy-3-methylbutanol, were identified as discriminant volatiles of AD beans, two others, namely, 2-ethylhexanol and 1-octanol, were identified for FD beans. For VD beans, there was no alcohol as a major compound compared to others. In previous studies, 2-hexanol, ethanol, 2-methylpropanol, 3-methylbutanol and 1-octanol have been reported in cooked common beans [26,40,41]. The decrease in alcohol compounds could be explained by evaporation after the cooking step or conversion to another compound during subsequent drying [28]. However, as the threshold concentrations of alcohols are relatively high, their contribution to the flavor profile is not high [26].

Discriminant aldehydes were found in AD beans (2-ethyl-3-methyl-butanal, nonanal) and VD beans (4-ethyl-benzaldehyde). During the drying process, 3-methylbutanal is formed as the result of Strecker degradation of valine and (iso)leucine. Therefore, it is considered a typical compound of dried vegetables [42]. Additionally, the presence of the aliphatic aldehyde nonanal suggests the thermally induced oxidation of unsaturated fatty acids during processing [28,41]. Fatty acids serve as precursors of various volatile compounds, which greatly contribute to aroma profiles as fresh, green and fruity notes. Degradation of fatty acids can be induced by non-enzymatic oxidation, such as autoxidation and heat-induced oxidation. Since the beans were cooked before dehydration, enzymatic pathways were not likely to be the main results in the formation of fatty acid oxidation products. High-temperature processing is one of the factors that promote the oxidative breakdown of fatty acids [43]. Regarding benzaldehyde, this compound is also the product of Strecker degradation of the amino acid phenylalanine, which occurs as an important side chain pathway of the Maillard reaction [43,44].

Out of the volatile discriminant compounds, a few ketones, namely, 3,5-octadien-2-one and methyl-isobutyl-ketone, were found in freeze-dried beans. Ketones are formed as the result of oxidative degradation of unsaturated fatty acids during thermal processing [45]. Regarding alkanes, an increase in their concentrations in functionalized beans was observed: n-hexane (AD beans), 2,9-dimethyl-decane, 2,3,6,7-tetramethyl-octane (VD beans), pentane and 3,3-dimethyl-hexane (FD beans). The generation of alkanes occurs due to the lipid oxidative decomposition from which they are derived [46]. Alkanes are not responsible for food flavor; however, their impact on volatility, as well as the flavor-imparting characteristic of other volatile compounds, cannot be neglected [26].

To obtain more insight into the influence of processing on volatile profiles of rehydrated dried beans, changes in key volatile compounds were further considered for in-depth analysis. Those key components responsible for the characteristic flavor of specific products are established based on their relative quantities and/or odor threshold. The lower the odor threshold concentration, the greater the influence that the compound exerts on the quality and uniqueness of the food flavor [47]. From the literature, hexanal, hexanol and dimethyl sulfide are recognized as chief volatile compounds of cooked red beans [26,39,40,46,48]. While hexanal provides typical green, fruity, grassy notes, dimethyl sulfide is responsible for the fruity, sulfury, cabbage-like aroma. Hexanol was reported to contribute aroma notes of banana, flower, grass and green to the bean flavor [39,46].

**Figure 6:**
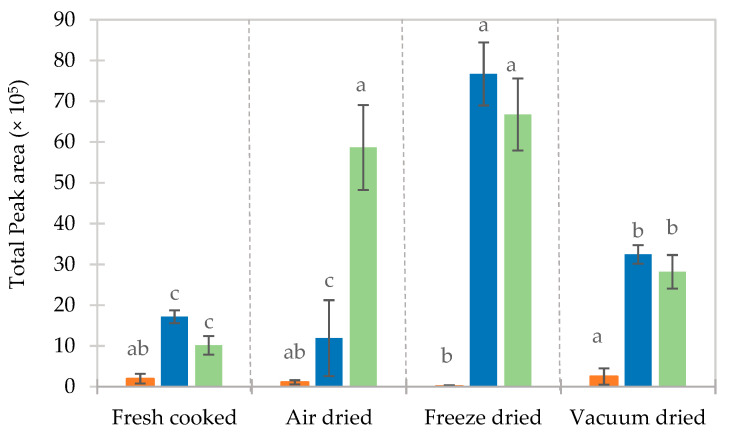
The influence of the processing method on the retention of key volatile compounds: dimethyl sulfide (■), 2-ethyl-1-hexanol (■) and hexanal (■), in rehydrated pre-cooked beans. Parameters not sharing the same letters are significantly different (*p* < 0.05).

The influence of the processing method on the retention of key volatile compounds of dehydrated pre-cooked beans is presented in Figure 6. Concerning dimethyl sulfide and 2-ethyl-1- hexanol, their total peak area in freshly cooked beans and AD beans showed an insignificant difference (*p* > 0.05). FD beans had the highest abundance of 2-ethyl-1-hexanol and hexanal, whereas 2-hexanol was only found in freshly cooked beans (the figure is not shown). The higher peak area of key compounds in FD foods was expected as the low-temperature application minimizes the thermal degradation of flavor compounds [49].

## 4. Conclusions

This study concludes that it is not only the type of drying process that influences the rehydration characteristics of dried beans but also multiple other parameters such as the temperature of rehydration, nature of pores and state of water-imbibing constituents. Limited damage to the cell wall and gelatinized starch during drying was strongly responsible for the high rehydration ability irrespective of the drying technique. The rehydration time, on the other hand, was strongly influenced by the process-induced microstructure, with a high porosity and higher temperature of rehydration favoring a shorter rehydration duration. Freeze drying created beans with the fastest rehydration rate, but the post-rehydration texture suggested damage to the cellular network during the freeze drying. However, the fragility of the FD product coupled with the high cost associated with drying can be a drawback. The quality of dried beans depends not just on the nutritional content but also on the functionalities such as the sensory characteristics (flavor, color and texture) and usage attributes (convenience/rehydration). The presence of key volatile compounds and the retainment of the color and textural attributes of the dried beans upon rehydration positively point towards the potential of the chosen drying techniques, with AD and VD being cost-effective too. Drying at low temperatures (<60 °C) is suggested for achieving optimal functionality in the dried beans upon rehydration. Therefore, the understanding of the process and/or structure remains a major factor controlling functionality, as demonstrated, for the first time, in this study.

## Figures and Tables

**Table 1:** Parameter estimates (parameter ± standard error) of activation energy (*E_a_*, kJ/mol), *K_ref_* (min %^−^ and min for Peleg and Weibull, respectively), effective diffusivity (*D_eff_*_0_, m^2^/s) and the estimated geometric factor.

	Peleg Model		Weibull Model		Fick’s Diffusion Model		
	*E_a_* (kJ/mol)	*K_ref_* (min %^−^)	*E_a_* (kJ/mol)	*K_ref_* (min)	*E_a_* (kJ/mol)	*D_eff_*_0_ × 10^−8^ (m^2^/s)	Geometric Factor
**VD**	13.32 ± 1.76	0.020 ± 0.00	14.37 ± 1.31	4.52 ± 0.06	14.70 ± 1.31	6.533 ± 0.0766	6.53 ± 0.07
**AD**	4.36 ± 3.01	0.017 ± 0.00	6.90 ± 1.92	4.77 ± 0.11	8.99 ± 1.54	5.53 ± 0.0704	5.53 ± 0.07
**FD**	2.05 ± 1.58	0.002 ± 0.00	2.86 ± 1.18	0.61 ± 0.008	3.45 ± 1.59	6.093 ± 0.0912	6.09 ± 0.09

**Table 2:** Color parameters (L*, a*, b* and ΔE*), temperature of gelatinization/melting (T_G_) and degree of retrogradation (DR%) of dried beans, and color change (ΔE_r_*), hardness (N), Y and RRM of rehydrated beans (mean ± standard deviation) ^1^.

	Control	Air-Dried	Vacuum-Dried	Freeze-Dried
L*	24.42 ± 0.89 ^b^	20.72 ± 0.93 ^a^	19.24 ± 0.58 ^a^	31.32 ± 1.55 ^c^
a*	18.31 ± 0.53 ^c^	8.76 ± 0.45 ^a^	9.10 ± 0.13 ^a^	12.0 ± 0.83 ^b^
b*	8.65 ± 0.74 ^c^	2.73 ± 0.24 ^a^	2.70 ±0.02 ^a^	4.50 ±0.47 ^b^
ΔE*	-	11.85 ± 0.68 ^a^	12.14 ± 0.35 ^a^	10.26 ± 1.14 ^a^
ΔE_r_*	-	0.96 ± 0.05 ^a^	3.55 ± 1.04 ^ab^	2.20 ± 0.99 ^b^
H (N)	64.82 ± 11.40 ^a^	62.90 ± 12.86 ^a^	70.50 ± 13.50 ^a^	48.21 ± 9.74 ^b^
T_G_ (°C)	79.74 ± 0.06 ^b^	57.57 ± 0.71 ^a^	64.76 ± 0.74 ^c^	58.33 ± 0.54 ^a^
DR (%)	-	44.79 ± 1.62 ^b^	28.89 ± 1.47 ^a^	27.04 ± 0.70 ^a^
Y	-	2.30 ± 0.013 ^a^	2.28 ± 0.043 ^a^	2.56 ± 0.001 ^b^
RRM	-	0.96 ± 0.008 ^a^	0.93 ± 0.029 ^a^	1.12 ± 0.001 ^b^

Values in the row not sharing the same letter are significantly different (*p* < 0.05).

**Table 3:** Discriminant volatile headspace components for each class selected by the VID procedure (>ǀ0.80ǀ). The compounds are listed in increasing order of VID, with a positive value indicating a higher concentration and vice versa. RI stands for retention index. Compounds in bold and italic are volatiles that were absent in freshly cooked beans.

VID	Compound	RI	Chemical Group
**Freshly cooked beans**			
0.994	2-Acetyl-3,5-dimethylfuran	1589	Furan compound
0.993	1-[2-(1-Methylethylidene) cyclopropyl] ethanone	1341	Ketone
0.992	2,4,4-Trimethyl-3-(3-methylbutyl) cyclohex-2-enone	1760	Ketone
0.989	2-ethanol	1719	Alcohol
0.987	4-methyl-3-penten-2-one	1134	Ketone
0.979	Tetramethylfuran	1650	Furan compound
0.974	3-(2,4-Dinitrophenyl) propanoic acid	1323	Organic acid
0.965	Octyl ester cyclopentane carboxylic acid	1453	Ester
0.956	1-Oxacyclododecan-2,8-dione	1330	Ketone
0.950	5-methyl-5-(1-methylethyl)-3-Heptyne-2,6-dione	1434	Ketone
0.943	5-Hepten-2-one	1330	Ketone
0.942	2-Hydroxy-3,5-dimethylcyclopent-2-en-1-one	1319	Ketone
0.905	4-Hydroxybutyric acid	1657	Organic acid
0.903	2-methyl-1-propanol	1093	Alcohol
0.899	Methanethiol	867	Sulfur compound
0.892	Ethanol	954	Alcohol
0.878	3-methylfuran	932	Furan compound
0.866	1-(2,4,6-trimethylphenyl)- ethanone	1870	Ketone
0.864	2,4-Dimethylfuran	979	Furan compound
0.850	4-hydroxy-4-methyl-2-pentanone	1373	Ketone
0.841	2-methyl-butanoic acid	1685	Organic acid
0.839	2-Hexanol	1231	Alcohol
0.826	2H-1-Benzopyran derivative (Edulan I)	1496	Benzopyran derivatives
0.807	Ethylhexyl benzoate	1728	Ester
**Air-dried beans**			
0.977	n-Hexane	859	Alkane
0.965	Nonanal	1399	Aldehyde
0.958	***o-Fluoroacetophenone***	1123	Ketone
0.900	***Methyl cyclopentane***	866	Cycloalkane
0.898	***Pentanoic acid***	1853	Organic acid
0.871	***2-phenyl-ethanol***	1924	Alcohol
0.869	2-ethyl-3-methyl-butanal	1063	Aldehyde
0.823	***3-Methoxy-3-methylbutanol***	1447	Alcohol
0.767	***4-Hydroxybenzyl alcohol***	1891	Alcohol
**Vacuum-dried beans**			
0.951	***4-ethyl-benzaldehyde***	1736	Aldehyde
0.945	Limonene	1203	Terpene
0.841	***2,9-dimethyl-decane***	1033	Alkane
0.818	***2,3,6,7-tetramethyl-octane***	1067	Alkane
−0.816	Dihydrobarrelene	1132	Alkene
**Freeze-dried beans**			
0.946	Pentane	855	Alkane
0.944	***3,5-Octadien-2-one***	1535	Ketone
0.938	2-ethyl-1-hexanol	1502	Alcohol
0.862	***3,3-dimethyl-hexane***	1200	Alkane
0.807	1-Octanol	1566	Alcohol
0.800	***Methyl Isobutyl Ketone***	1008	Ketone
−0.942	Acetic acid	1469	Organic acid

## Data Availability

The data used for the figures is available on 10.5281/zenodo.5112772.

## Supplementary Materials

The following are available online at https://www.mdpi.com/article/10.3390/foods10071665/s1, Table S1: Parameter estimates and goodness of fit of the empirical rehydration models, Table S2: Parameter estimates of the diffusion model, Figure S1a: Parity plot (1), and residues versus time (2) of Weibull model for rehydration data fitting of (a) air-dried, (b) vacuum-dried and (c) freeze-dried beans. Figure S1b: Parity plot (1), and residues versus time (2) of the diffusion model for rehydration data fitting of (a) air-dried, (b) vacuum-dried and (c) freeze-dried beans.
