# Supplementary material for: The Impact of Drying and Rehydration on the Structural Properties and Quality Attributes of Pre-Cooked Dried Beans
Source: Foods. 2021 Jul 19;10(7):1665. doi: 10.3390/foods10071665 (PMC8305296; doi:10.3390/foods10071665)
Supplement: Supplementary file 1 [file foods-10-01665-s001.zip › foods-1294042-supplementary.pdf]

Supplementary materials

Table S1. Parameter estimates and goodness of fit of the empirical rehydration models

Models	Parameters	Drying methods											
		Vacuum-dried				Air-dried				Freeze-dried			
		70 °C	80 °C	90 °C	100 °C	70 °C	80 °C	90 °C	100 °C	70 °C	80 °C	90 °C	100 °C
Peleg	K_1	0.028	0.024	0.020	0.016	0.023	0.021	0.020	0.017	0.002	0.002	0.002	0.002
	K_2	0.008	0.007	0.006	0.006	0.007	0.007	0.006	0.007	0.006	0.006	0.005	0.005
	R^2	1.000	0.998	1.000	0.999	0.996	0.997	0.995	0.998	0.999	0.999	0.999	0.999
	RMSE	0.047	0.031	0.014	0.013	0.060	0.058	0.077	0.027	0.406	0.390	0.391	0.410
	$\chi 2$	0.256	0.096	0.016	0.012	0.337	0.348	0.466	0.060	0.337	0.348	0.466	0.060
Weibull	β	0.576	0.634	0.724	0.718	0.512	0.520	0.487	0.659	0.552	0.581	0.643	0.618
	α	5.705	5.366	4.561	3.955	5.335	5.004	4.837	4.551	0.630	0.645	0.650	0.587
	R^2	0.996	0.999	1.000	0.999	0.994	0.994	0.992	0.998	0.998	0.998	0.995	0.999
	RMSE	0.022	0.014	0.007	0.010	0.026	0.025	0.030	0.016	0.014	0.013	0.024	0.013
	$\chi 2$	0.028	0.009	0.001	0.002	0.023	0.026	0.041	0.009	0.005	0.005	0.030	0.005
Exponential	k	0.367	0.345	0.333	0.373	0.424	0.433	0.465	0.368	1.290	1.290	1.319	1.390
	n	0.576	0.634	0.724	0.718	0.512	0.520	0.487	0.659	0.552	0.581	0.643	0.618
	R^2	0.996	0.999	1.000	0.999	0.994	0.994	0.992	0.998	0.998	0.998	0.995	0.999
	RMSE	0.022	0.014	0.007	0.010	0.026	0.025	0.030	0.016	0.014	0.013	0.024	0.013
	$\chi 2$	0.028	0.009	0.001	0.002	0.023	0.026	0.041	0.009	0.005	0.005	0.030	0.005
First order	H	0.208	0.219	0.246	0.283	0.190	0.174	0.203	0.200	1.638	1.599	1.590	1.720
	R^2	0.936	0.962	0.982	0.980	0.895	0.886	0.881	0.901	0.935	0.947	0.964	0.958
	RMSE	0.090	0.074	0.051	0.053	0.111	0.105	0.118	0.069	0.082	0.075	0.068	0.069
	$\chi 2$	0.903	0.466	0.176	0.155	1.014	1.050	1.350	0.356	0.413	0.370	0.268	0.295

Table S2. Parameter estimates of the Diffusion model

	**D_{eff} x 10^{-8} (m^2/s)**	***Geometric factor***	***R^2***	***RMSE***	***$\chi 2$***
Vacuum-dried					
70 °C	0.837 ± 0.026	5.847 ± 0.081	0.999	0.014	0.013
80 °C	1.066 ± 0.034	6.396 ± 0.095	0.999	0.015	0.010
90 °C	1.543 ± 0.036	7.166 ± 0.086	0.999	0.011	0.006
100 °C	1.768 ± 0.053	7.151 ± 0.112	0.999	0.014	0.006
Air-dried					
70 °C	0.707 ± 0.021	5.304 ± 0.070	0.999	0.013	0.006
80 °C	0.780 ± 0.021	5.353 ± 0.064	0.999	0.011	0.005
90 °C	0.733 ± 0.031	5.122 ± 0.097	0.998	0.018	0.015
100 °C	1.347 ± 0.044	6.618 ± 0.112	0.999	0.016	0.012
Freeze-dried					
70 °C	6.975 ± 0.390	5.646 ± 0.147	0.997	0.022	0.019
80 °C	7.605 ± 0.385	5.936 ± 0.143	0.997	0.020	0.015
90 °C	9.163 ± 0.673	6.539 ± 0.240	0.994	0.031	0.043
100 °C	9.507 ± 0.440	6.299 ± 0.147	0.998	0.019	0.011

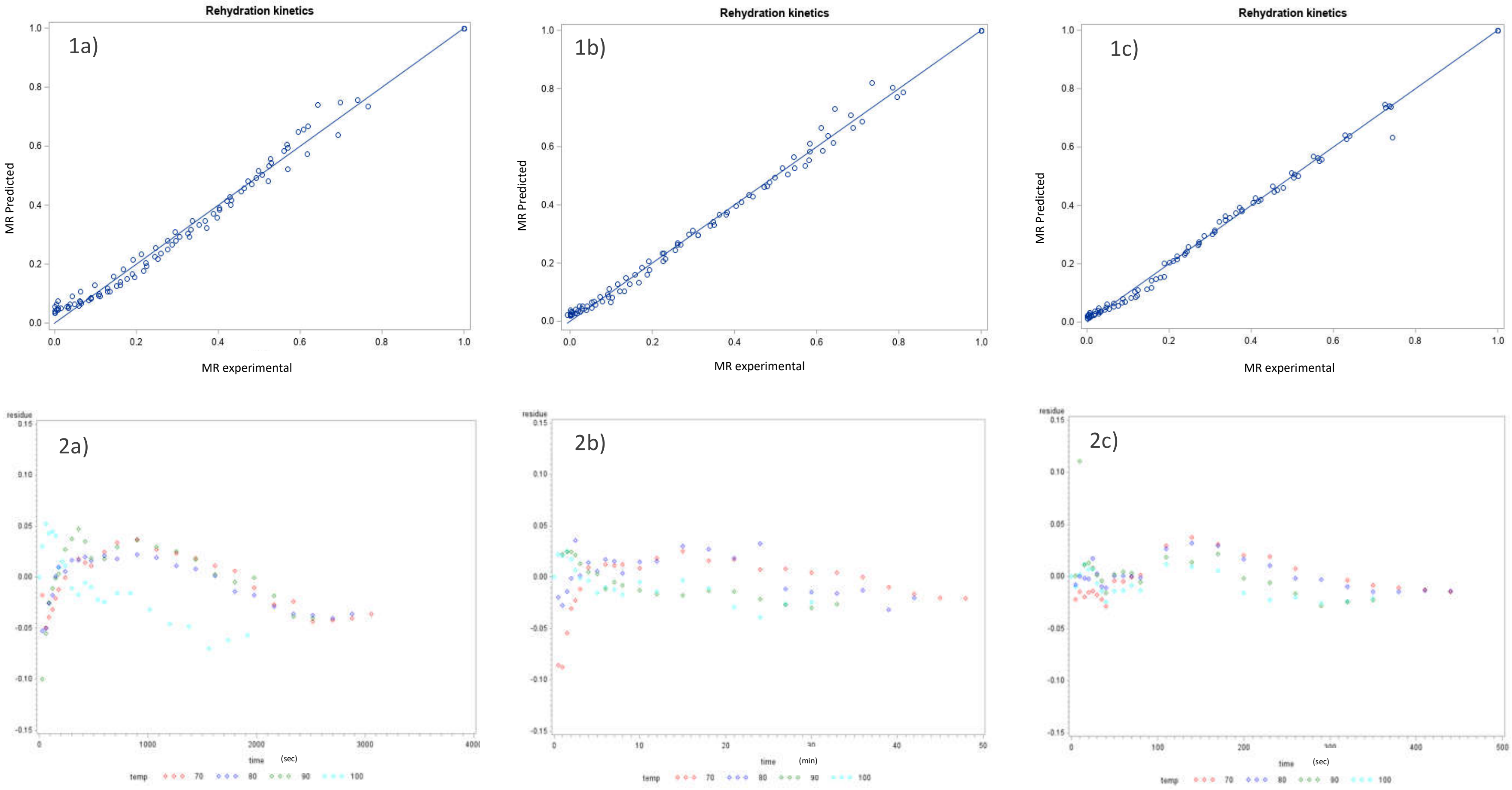

Figure S1a: Parity plot (1) and Residues versus time (2) of Weibull model for rehydration data fitting of (a) air-dried, (b) vacuum-dried and (c) freeze-dried beans.

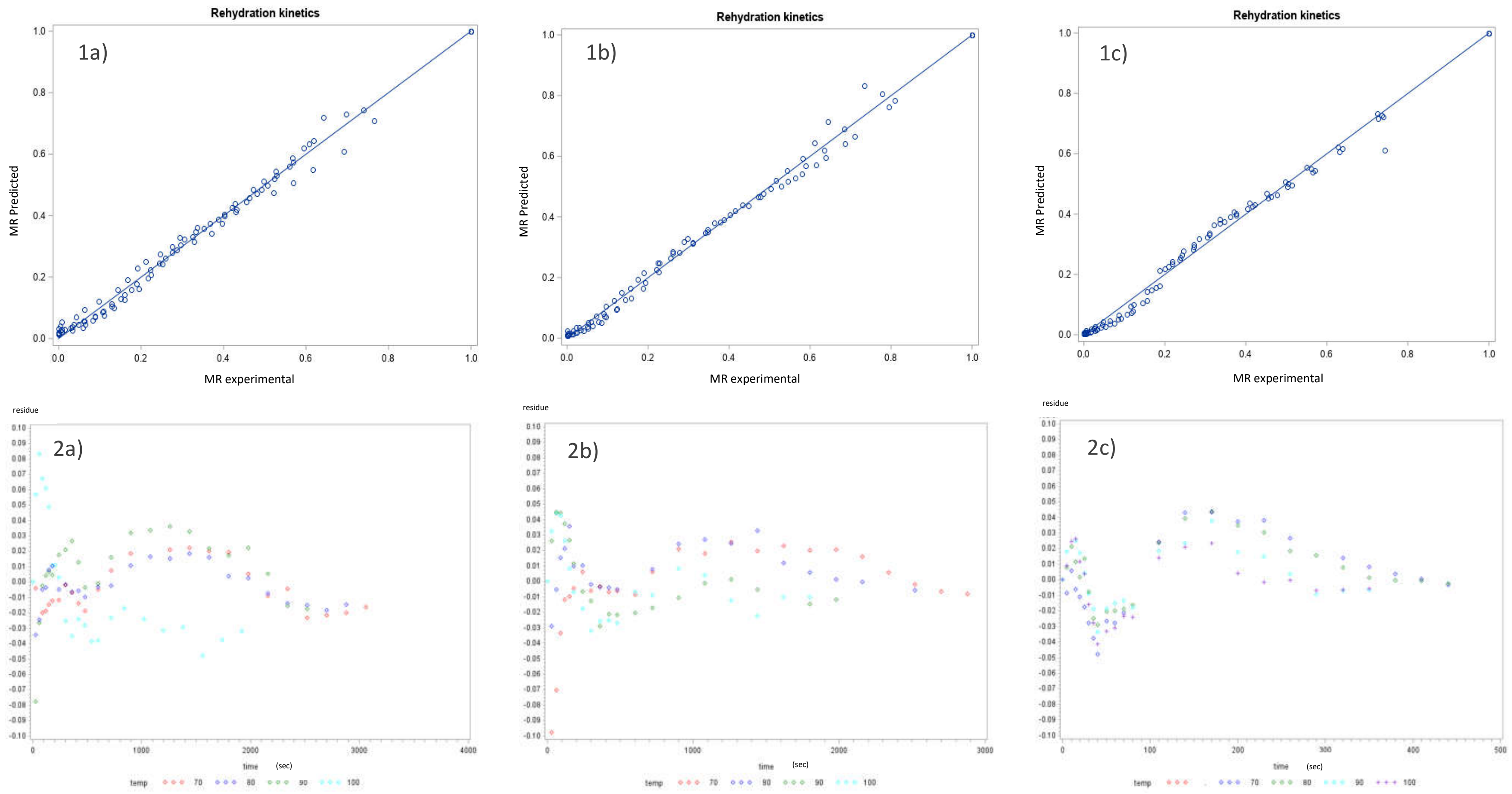

Figure S1b: Parity plot (1) and Residues versus time (2) of the diffusion model for rehydration data fitting of (a) air-dried, (b) vacuum-dried and (c) freeze-dried beans
